# A color extraction algorithm by segmentation

**DOI:** 10.1038/s41598-023-48689-y

**Published:** 2023-12-02

**Authors:** QingE Wu, Zhenggaoyuan Fang, Zhichao Song, Hu Chen, Yingbo Lu, Lintao Zhou, Xiaoliang Qian

**Affiliations:** School of Electrical and Information Engineering, Zhengzhou University of Light Industry, Zhengzhou, 450002 China

**Keywords:** Computer science, Information technology

## Abstract

The segmentation and extraction on color features can provide useful information for many different application domains. However, most of the existing image processing algorithms on feature extraction are gray image-based and consider only one-dimensional parameters. In order to carry out a fast and accurate color feature extraction, this paper proposes a color extraction algorithm by segmentation that is called a color extraction algorithm This algorithm is compared under different color distribution situations, and the extraction effect on color is also shown by the combination of the segmentation and feature extraction algorithms. Experimental results show that such segmentation algorithm has some advantages for color segmentation. In the fuzzy color image preprocessing, this paper gives the location method of region of interest. Moreover, compared with other existing extraction algorithms, the presented segmentation extraction algorithm in this paper not only has higher accuracy, shorter extraction time and stronger anti-interference ability, but also has better effect on more divergent color edge. Experimental evaluation of the proposed color extraction algorithm demonstrates dominance over existing algorithms for feature extraction. These researches in this paper provide a new way of thinking for color feature extraction by segmentation, which has an important theoretical references and practical significance.

## Introduction

For the extraction of characteristic points, the existing literature was shifted toward color images with three-dimensional parameters additional work is still required. A widespread use of color images in more and more application domains as they offer more valuable information. For example, during the surgery the operation enhancing is done with the help of images. Initially, the success rate of operations was not too high, which can be attributed to the use of only gray-scale images. However, recent surgery is enhanced using color Doppler ultrasound, such as a color picture or projection, and the surgery success rates have improved significantly. To further improve surgical success rate as well as other application domains, new image processing algorithms on feature extraction for edges and corner points of color images should be developed.

Researchers in image processing focus on identifying content edges. The commonly used methods for image edge detection include Roberts operator, Prewitt operator, Kirsch operator, Laplace operator, Sobel operator, Canny operator, etc. These algorithms are widely used in various fields such as roughness recognition^1^ and watermarking^2^. H T S K et al.^3^ proposed an algorithm to optimize the interruption edge of the Canny operator. To improve the application of Sobel operator, Rakesh et al.^4,5^ propose an improved algorithm that utilizes weighted guided image filtering and improved adaptive threshold. By combining fractional order differentiation, Saeed et al.^6^ improved the Prewitt operator and Mishra et al.^7^ proposed an edge analysis method for gradient detection. Wang et al.^8^ proposed a subpixel edge extraction method. These image algorithms, combined with further noise reduction processing^9–11^, are applied in engineering assignments^12,13^. However, the loss of color and features in image extraction was still significant. The existing image segmentation methods are mainly divided into the following categories: threshold-based segmentation methods, region-based segmentation methods, edge-based segmentation methods, and segmentation methods based on specific theories. Xie et al.^14^ proposed a non-local active contour model for a fast unsupervised image segmentation algorithm for texture images. Lo et al.^15,16^ improved the image segmentation algorithm through wavelet transform and interactive segmentation methods. Long et al.^17–19^ proposed algorithms for image segmentation through model analysis. Li et al.^20^ denoised the image segmentation model through regularization. Color segmentation, as a relatively complex image segmentation content, faced problems such as unclear edges and weak discrimination even after algorithm improvements and noise reduction^21–24^.

To resolve identified problems related to valuable information on color images, this paper presents a color extraction algorithm by segmentation of three-dimensional parameters of color images for extraction of edges and corner points. In fact, the color extraction algorithm by segmentation studied in this paper is mainly composed of three parts, i.e., feature segmentation including image denoising and color feature segmentation, image enhancement, color feature extraction including adjustment of gray, extraction of edge contour, detection and extraction of feature points. Specifically, this paper gives the comparison and experiment of different color distributions. Finally, the proposed segmentation extraction is compared with common image analysis operators, and the application prospects under different conditions are shown.

## Feature segmentation algorithm

### Denoising for image

Because of the image acquisition system, different physical phenomena such as illumination cannot be completely evenly distributed, and for many other reasons, the obtained edge intensity of the image is different. Moreover, in real-world situations, image data is often contaminated by noise. The scenery features are mixed, so it makes subsequent interpretation very difficult. To achieve an accurate grasp of the picture intent, it needs to study a target recognition method that can not only detect the non-continuity of intensity but also determine their exact position. It needs to develop new uncertainty processing methods and algorithms to solve such problems. Here, a fuzzy operator for image denoising is given as follows:

Assume $F:R^{n} \to R$, if $F(a_{1} ,a_{2,} \cdots ,a_{n} ) = \sum\limits_{j = 1}^{n} {\omega_{j} } b_{j}$, where $\omega = (\omega_{1} ,\omega_{2} , \cdots \omega_{n} )$ is an $n$-dimension vector associated with the function $F$, $\omega_{j} \in \left[ 0 \right.,\left. 1 \right]$, $j \in \left\{ {1,2, \cdots } \right.\left. n \right\}$, $\sum\limits_{j = 1}^{n} {\omega_{j} } = 1$, and $b_{j}$ is the $j$th large element in a data set $(a_{1} ,a_{2} , \cdots ,a_{n} )$, $R$ is the set of real number, the function $F$ is called $n$-dimension ordered weighted average operator (OWA operator).

The obvious feature of OWA operator is that it can firstly reorder the given decision-making data $(a_{1} ,a_{2} , \cdots ,a_{n} )$ in descending order and obtain a new data $(b_{1} ,b_{2} , \cdots ,b_{n} )$, and aggregate the new data by the given weight vector. The weight value $\omega_{j}$ has no relationship with the element $a_{j}$, it's only related to the $j$th position in the aggregate process.

The OWA operator is the aggregation method of multiple attribute decision making between the maximum and minimum operator, i.e., assumed $$\omega = \left( {0,\frac{1}{n - 2}, \cdots ,\frac{1}{n - 2},0} \right)$$. $$F\left( {a_{1} ,a_{2} , \cdots a_{n} } \right) =$$ $$\sum\limits_{j = 1}^{n} {\omega_{j} b_{j} }$$$$= \frac{1}{n - 2}\sum\limits_{j = 2}^{n - 1} {b_{j} }$$. That is, this method removed the maximum and minimum values, and did the arithmetic average to the rest.

**Figure 1:**
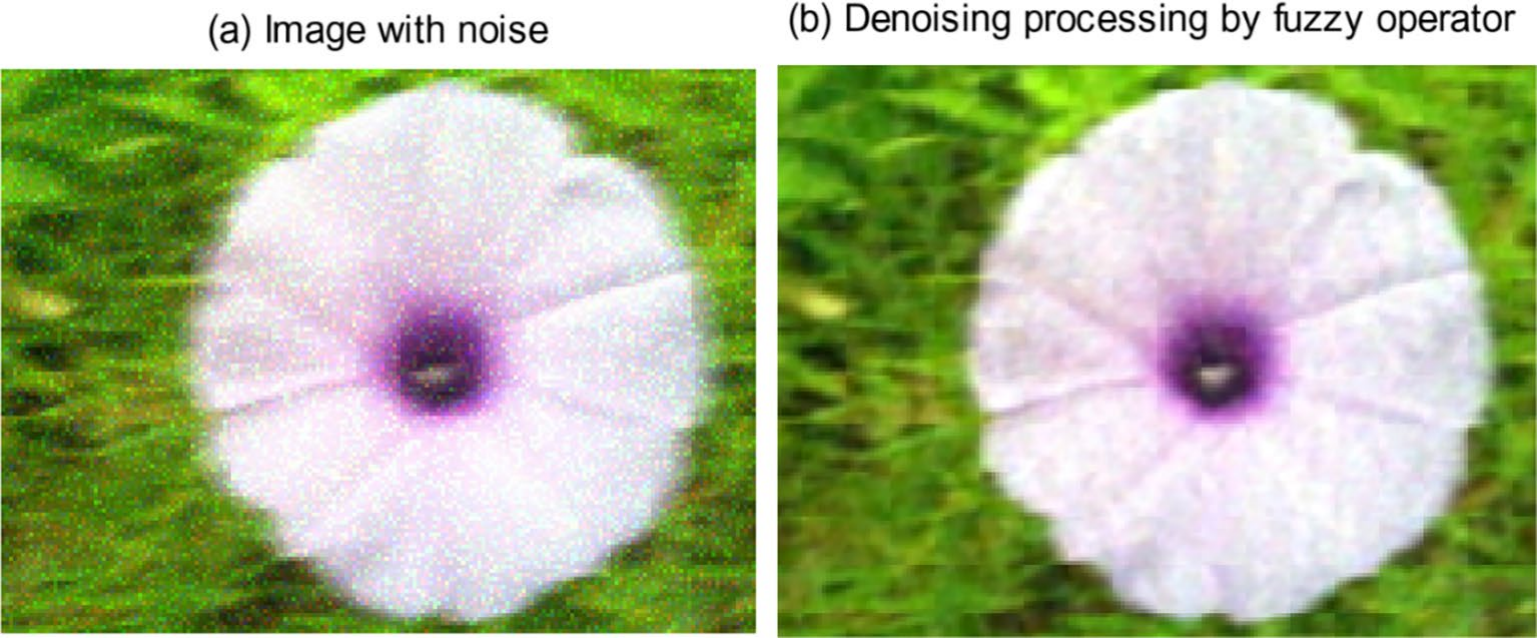
Denoising processing to fuzzy image. (**a**) Image with noise. (**b**) Denoising processing by fuzzy operator.

In some cases, the acquired most of images are fuzzy. In addition to objective reasons, there are also some subjective reasons to cause the images to be fuzzy, such as images were dissevered, soiled, etc. For the fuzziness in these images, this paper uses the fuzzy approach to process fuzzy signals based on uncertainty factor classification and influence analysis and presents a fuzzy signal processing method and algorithm that can improve image quality, as shown in Fig. 1.

The threshold value is used to smooth image processing and eliminate the fuzziness of the image.

### Color feature segmentation

We propose a probabilistic algorithm for segmentation to color images. This algorithm is based on the probability that the color of the homochromy pixel possesses in the entire region of interest (ROI). Its specific definition is as follows:

#### Definition 1

Divide the ROI of a color image. Calculate the number of pixels in ROI is $n$, and the number of pixels of a single color is $m$. Label the single color as ${\text{l}}_{c}$. Then the calculation of percentage of the single color ${\text{l}}_{c}$ in the ROI is called a probabilistic algorithm, and is defined as:

$$ \Pr \left( {{\text{l}}_{c} } \right) = \frac{m}{n} $$

This algorithm relies on the proportion that the pixel occurs in ROI. The ROI is chosen by the requirements of the target or testing. The ROI can be automatically chosen however it can be also manually chosen. For the ROI of color, the selection of an ROI is done through the value of hue. In the following subsections, we present selection algorithms for ROI.

**Figure 2:**
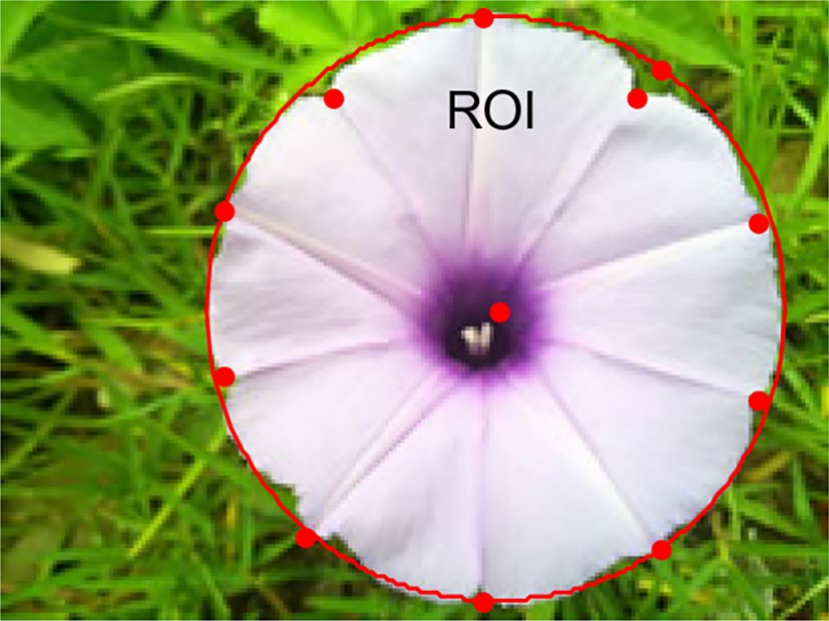
ROI chosen by computing the number of pixels of some colors.

If the composition of the color of the image is clear, the luminance value of each pixel is counted. The number of pixels of some colors can be computed by the luminance value. The counted pixels of each color are marked, respectively, and then the ROI can be marked out. In the simulation, we select the range of luminance value of the pixel as [200, 230], and use the "red" to mark the pixel. The simulation result is shown in Fig. 2, in which the red region is the delineated ROI.

## Enhancement to image

If a public threshold value is shared in all directions, then the enhancement method is called a public threshold enhancement. If the different threshold value is used in a different direction, the enhancement method is called an alone threshold enhancement.

According to experimental analysis, the weighting function $\omega$ = [0.95, 0.85, 0.8; 0.8, -8.1, 0.7; 0.85, 0.95, 1.2] is used to adjust the image $I$, and the image is adjusted as follows:

$$ G = \omega \otimes I $$

Then, the image smoothing is carried out by the 5 layers coefficient decomposition of the wavelet haar, and then the image is enhanced by using the threshold to sharpen it. By this proposed weight segmentation enhancement method for image, the quality of the color image is improved, and its clarity degree is also improved.

**Figure 3:**
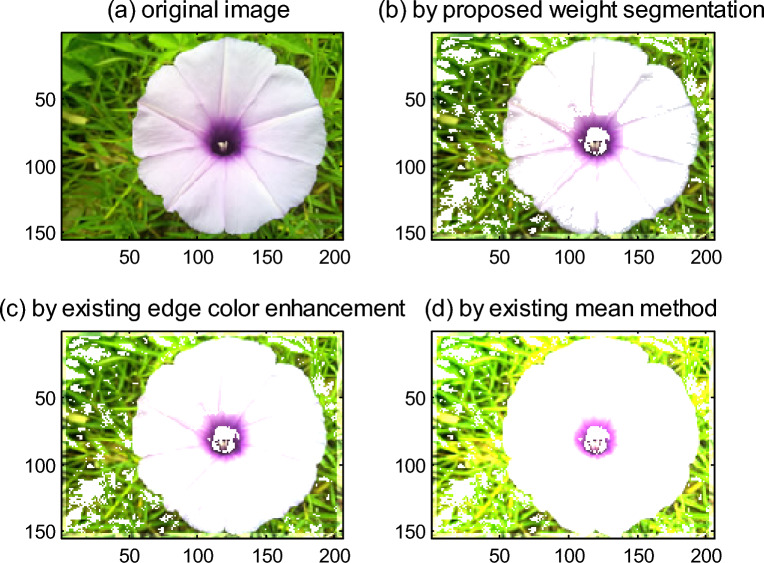
Comparison of the proposed weight segmentation and existing enhancement methods. (**a**) Original image. (**b**) By proposed weight segmentation. (**c**) By existing edge color enhancement. (**d**) By existing mean method.

To compare the proposed weight segmentation enhancement method with the existing edge color enhancement and color mean methods, the proposed enhancement method has a faster processing speed and a better effect than other enhancement methods for images, as is shown in Fig. 3.

## Feature extraction to color image

### Adjustment of gray

**Figure 4:**
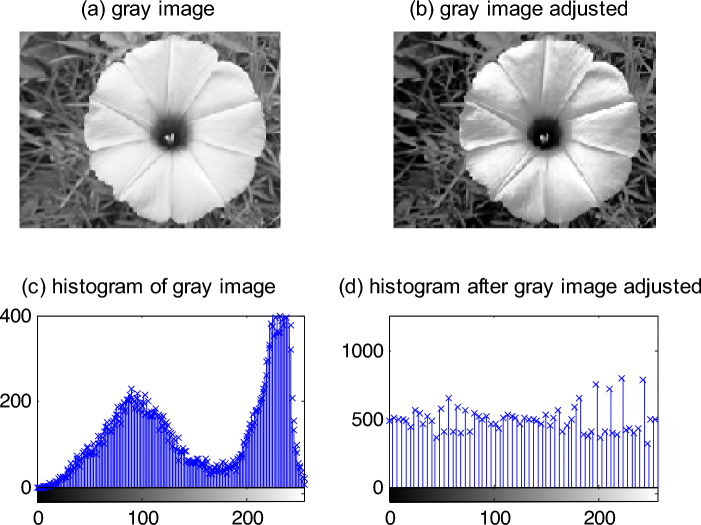
Adjustment of range of gray and histogram. (**a**) Gray image. (**b**) Gray image adjusted. (**c**) Histogram of gray image. (**d**) Histogram after gray image adjusted.

Firstly, convert the original RGB image into the gray image, and then adjust the range of the gray image from [0.3, 0.8] to [0, 1], to obtain a clearer texture pattern of the finger. Figure 4 shows the histogram of the image before and after adjustment. By the histogram, the grayscale range of distribution is uniform after the image transforms, therefore, an image with clearer texture information is obtained.

In Fig. 4, (a) is a gray image that the original RGB image is converted into. (b) is an image after the range of gray is adjusted. (c) is a histogram of the gray image. (d) is a histogram after the gray image is adjusted.

### Extraction of edge contour

The image border is the discontinuous reflection of local characteristics of an image, such as the mutation of gray level, colors, and texture, which marks the end of a region and the beginning of another region. For calculation simplicity, the first-order derivative or the second derivative is usually used to check the border of an image. It can be easy to detect the discontinuity of the gray level by taking advantage of the derivative method. The detection of borders can be realized by the convolution based on the spatial differential operators. Some smoothness and binarization processing should be done before the extraction of the border is implemented.

Assume the original fingerprint image is $f^{\prime}(x,y)$, the smooth filtering to $f^{\prime}(x,y)$ in a spatial domain can be carried out by using a low-pass filter $H$ and the output image is $g(u,v)$, as shown in equation.

1 $$ g(u,v) = \sum\limits_{x} {\sum\limits_{y} {f^{\prime}(x,y)H(u - x + 1,v - y + 1)} } $$

A relative complete image of fingerprint edge can be obtained after filtering. Then a binarization processing for image $g(u,v)$ is carried out by choosing a proper threshold value $h$. As shown in equation, the processed image is $f(x,y)$.

2 $$ f(x,y) = \left\{ {\begin{array}{*{20}c} {1,} & {g(u,v) > h} \\ {0,} & {g(u,v) \le h} \\ \end{array} } \right. $$

Then the edge detection for the binarization image can be carried out in image width and image height directions by making use of gray-level mutation.

### Detection and extraction of feature points

**Figure 5:**
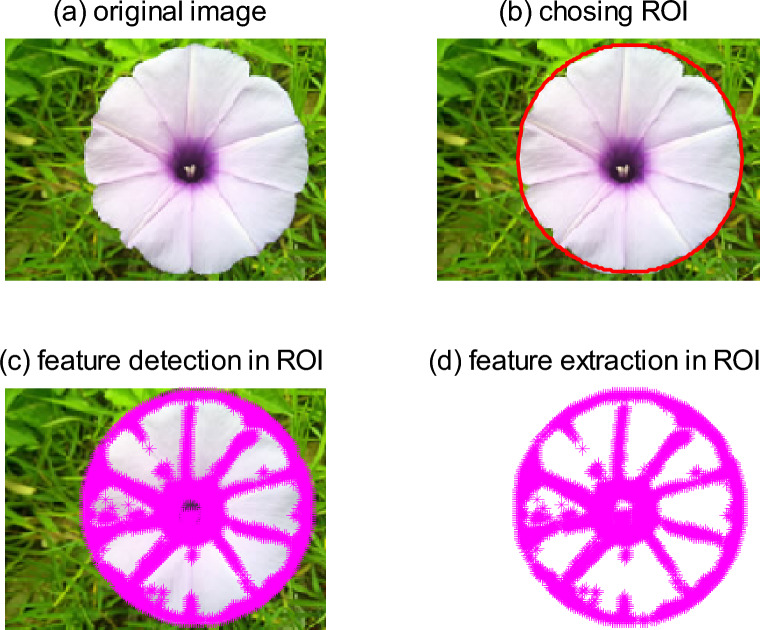
Detection and extraction to feature points based on above algorithm. (**a**) Original image. (**b**) Chosing ROI. (**c**) Feature detection in ROI. (**d**) Feature extraction in ROI.

The texture of the color image is clear through processing by the above step, so it is easy to better implement the detection of characteristic points and tracking them. According to the color level jump algorithm given above, it can not only achieve image edge detection but also can detect a corner point, endpoint, bifurcation points, etc. The detection of corner points based on the color level jump algorithm is shown in Fig. 5.

The characteristic points are the endpoints, corner points, bifurcation points, etc., which the papillary ridges form on the figure. The type of corner points detected by the color level jump algorithm is various, so it requires filtering the detected corner points. Select an enough small radius $r$, and then take the corner points as the center of circle to draw a circle. We detect the times of change of color level along this circle. If there are three sharp growth values or sharp reduction values of color level jump at a certain point, the point is a characteristic point.

## Comparison of proposed algorithm with the existing algorithms

In this paper, the proposed color segmentation algorithm and existing algorithms such as Robert, Sobel, Prewitt, Laplace, and Canny are compared by more experiments.

The experimental equipment for this comparison is a computer with Inter I7 6-core CPU, 32GB memory, and a 64-bit Windows 10 system. The experimental platform is Matlab2020 and Python3.7.

**Figure 6:**
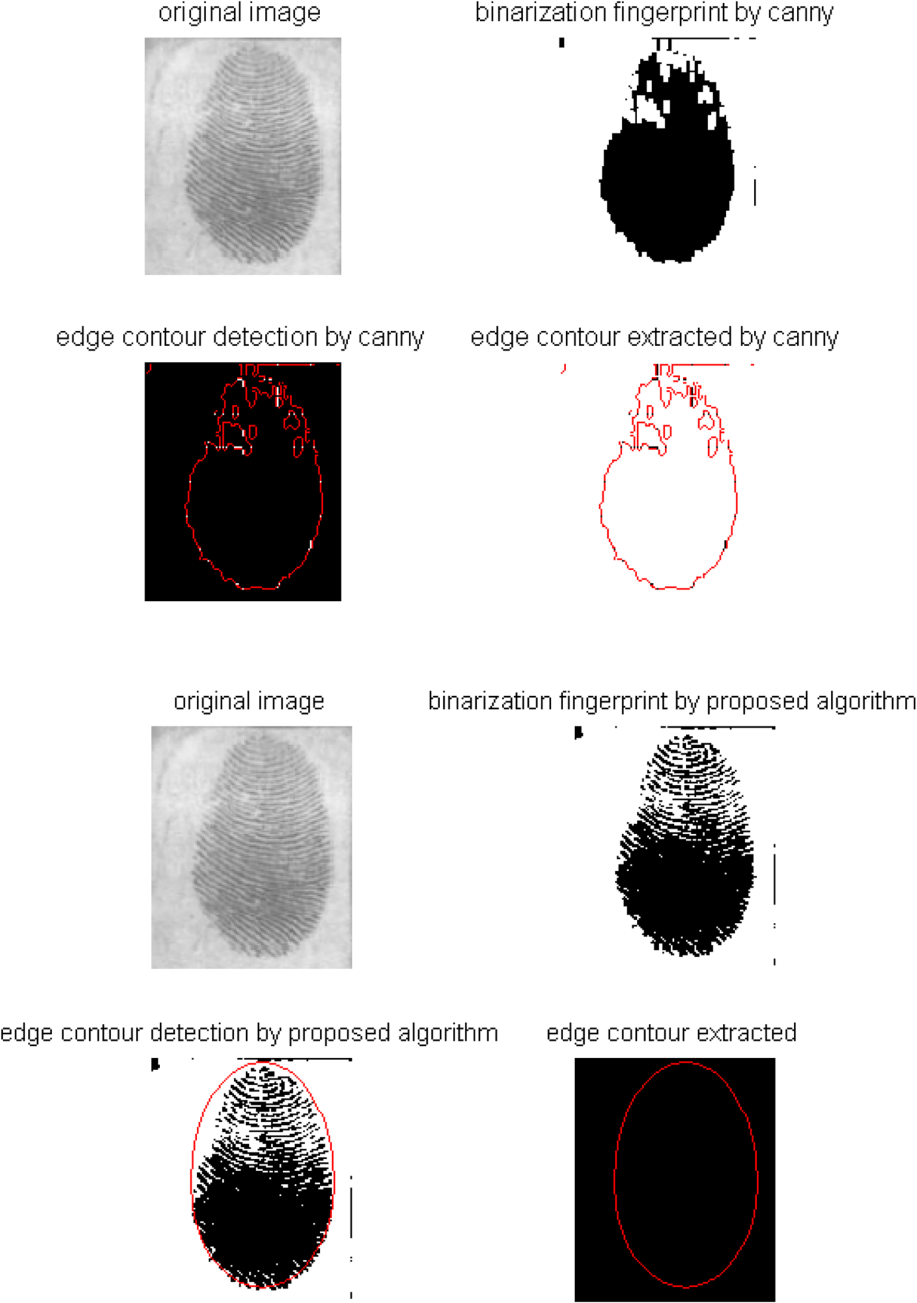
Comparison of fingerprint edge extraction between proposed and existing Canny.

Figure 6 gives the extraction process of fingerprint edge based on the mutation algorithm, and shows the comparison between this mutation algorithm and the existing best Canny extraction algorithm to fingerprint.

From Fig. 6, the marginal branches are many based on the Canny algorithm to the contour extracted. However, the edge contour effect extracted by the proposed extraction algorithm is good, because the extracted edge is smooth and the contours can basically locate the target region.

For feature extraction of color images, the extraction results which are given by two color feature extraction algorithms proposed in this paper and existing relevant extraction algorithms to edge and corner of color image are compared.

The simulation results show that the extraction effect of the pixel probabilistic algorithm to the color with ambiguous boundary is good, and its processing speed is fast. For a color image with size 362 × 500, the pixel probabilistic algorithm for the image processing is used, its processing time is 0.516 s, but its eliminating noise ability is relatively weak. However, the color segmentation algorithm is used to extract features of color, such as edges, corners, bifurcation points, and so on. The results show that its extraction effect is not only good, but also it can detect the edge direction information, as well as its eliminating noise ability is strong, and its processing speed is faster. For a color image with 362 × 500, its processing times are only 0.157 s. Moreover, this algorithm in use is flexible, which is because some thresholds, such as the threshold of the comparison function, threshold of edge response, and threshold of corner response, can be differently set in simulation based on actual situation and experimental analysis.

**Figure 7:**
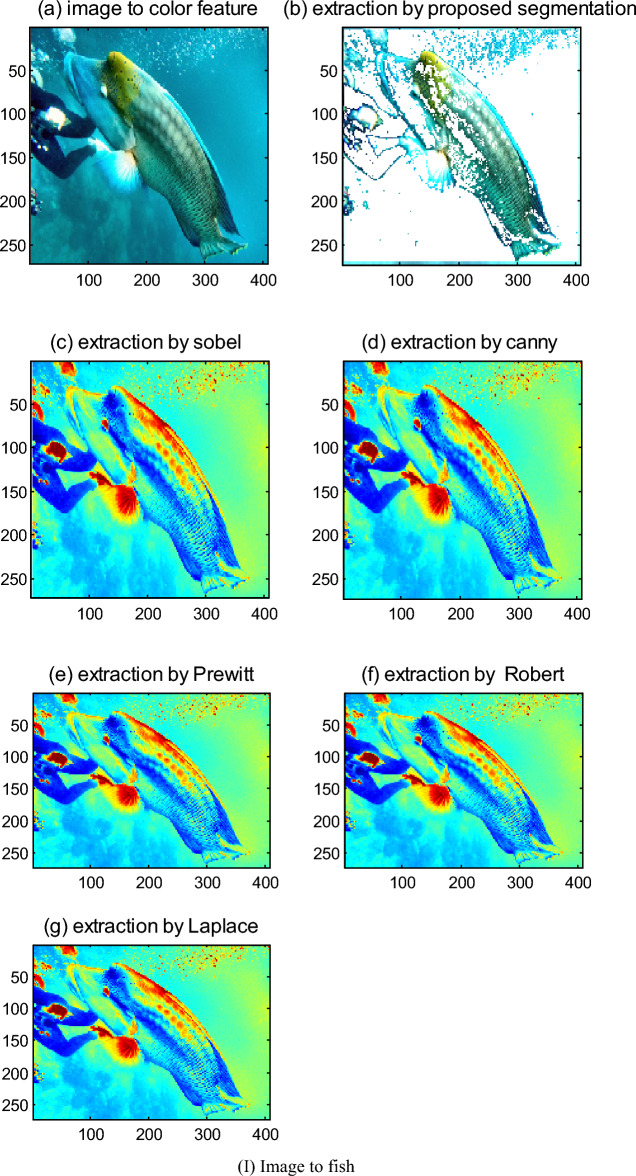

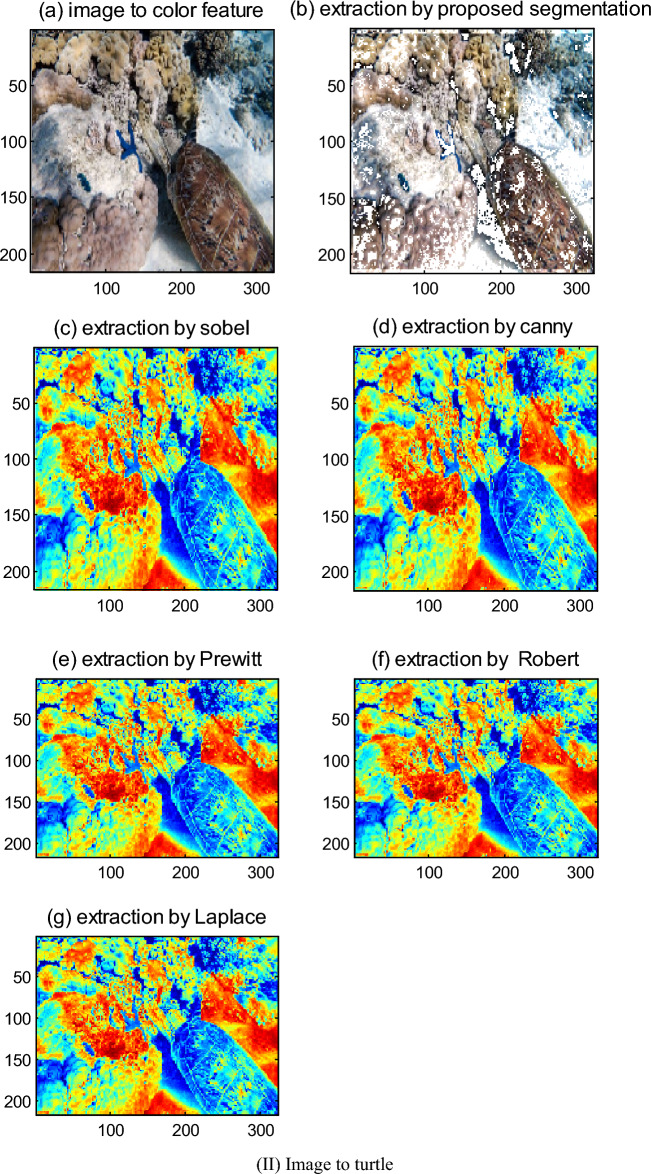
Comparisons of feature extraction of the proposed color segmentation and existing algorithms to color image. (I) Image to fish. (**a**) Image to color feature. (**b**) Extraction by proposed segmentation. (**c**) Extraction by Sobel. (**d**) Extraction by Canny. (**e**) Extraction by Prewitt. (**f**) Extraction by Robert. (**g**) Extraction by Laplace. (II) Image to turtle. (**a**) Image to color feature. (**b**) Extraction by proposed segmentation. (**c**) Extraction by Sobel. (**d**) Extraction by Canny. (**e**) Extraction by Prewitt. (**f**) Extraction by Robert. (**g**) Extraction by Laplace.

However, the classical Robert, Sobel, and Prewitt operators could not detect the edges of some straight lines, and neither could not detect the part edges of a circle. Though Gauss-Laplace and Canny operators could basically detect all the edges, their positioning effect is relatively poor, the edge pixels detected by them are wider, and the edges and corners extracted by them all were lost much. Their processing speed is slightly slow. For a color image with 362 × 500, the average processing time of the proposed algorithm is 0.157 s, however, Sobel, Laplace, and Prewitt operators are about 13.969 s, that of Robert is 0.75 s, that of Canny is 21.93 s. Moreover, their eliminate noise ability is weak. Simulation results are shown in Fig. 7bs–gs, and as are the original image.

To verify the color feature matching accuracy of the edge extraction algorithm proposed in this paper, the data is selected again in the data set for accuracy testing. Establish a standard template database.

300 different targets were randomly selected from the self-sampling image database. Choosing 3 randomly from 10 target images of each target to form a total of 500 target images.

A total of 300 target images were randomly selected from the three target images of each target to form the experimental target image database. The remaining 200 target images are from the test sample library.

Using the algorithm proposed in this paper to extract color features from 300 target images in the established target image database. A set of color feature vectors decomposed by 300 templates can be obtained. Each feature set contains 300 feature vectors. The extracted feature vectors are stored in the collected natural image archive as training samples.

The correctness of the color feature extraction is verified, and the verification steps are as follows:

Step 1. Using template transformation to decompose and extract the color of the target image in the test sample library, and obtain the color features of two-layer template decomposition. Then the obtained feature vector is matched with the samples in the image database.

Step 2. Matching the image color features to be tested with all sample colors in the image database, conducting 500 random selection and matching tests, and judging the vector features corresponding to the most similar colors as the test results.

Step 3. Performing 500 inspections for each image in the test sample database according to steps 1 ~ 2, record the number of correct inspections and incorrect inspections, and calculate the correct inspection rate.

From the result analysis, for each target image, the template transformation was selected in the simulation, and the experiments were repeated 20 times according to steps P1 to P3, and different numbers of samples were taken for each experiment. It is compared with Gabor and the statistical test algorithms that are widely used at present. The correct average extraction rate of the proposed segmentation algorithm for 500 simulations is 95.26%, however, that of the existing best algorithms is 91.25%.

**Figure 8:**
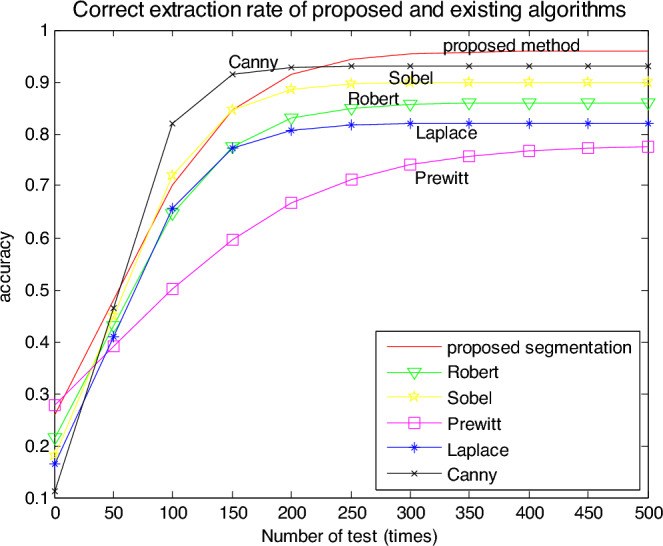
Comparison of extraction accuracy for the proposed and existing algorithms.

The proposed algorithm is not only effective in processing time but also improves the recognition accuracy by an average of about 4% compared with the existing best algorithms. The extraction accuracy of the several algorithms is shown in Fig. 8.

To evaluate the overall performance of each algorithm, we adopt the combination of quantitative analysis and qualitative analysis algorithms and synthesize comparisons based on several factors such as the correct color extraction rate, processing speed, anti-noise capability, loss of color, and so on. We evaluate the merits and demerits of the proposed algorithm and existing algorithms. Table 1 gives the results of a comprehensive comparison.

**Table 1:** Comprehensive comparison of several color extraction algorithms.

Algorithms	Correct color extraction rate	Processing speed (s)	Anti-noise capability	Loss of color	Acclimation
Proposed segmentation	0. 9526	0.157	Stronger	Less	Complex
Robert	0.8299	0.75	Weak	Much	Low noise
Sobel	0.8712	13.969	Weak	More	Low noise and gray changes gradually
Prewitt	0.7118	13. 897	Weak	Much	Low noise and gray changes gradually
Laplace	0.7711	21.61	Weak	Much	Low noise
Canny	0.9125	21.932	Little strong	Much	Little complex

From Table 1 and Fig. 8, the correct color extraction rate of the proposed color segmentation algorithm to color image is higher than that of existing algorithms, its processing speed is the fastest among several algorithms, its anti-noise ability is stronger, as well as the loss of color extracted by the algorithm is less than that by other algorithms. In a word, its robustness is the best.

## Conclusion

This paper studies the features of color images, presents the feature extraction of three-dimensional parameter color images, gives the extraction algorithms of edges and corners, as well as gives the comparison and experiment of the two algorithms under different color distributions. Finally, for feature extraction of color images, the extraction results which are given by the two proposed algorithms and existing extraction algorithms to the edge and corner of the color image are compared. For the similar color segment algorithm to extract the features of a color image, such as edges, corners, bifurcation points, and so on, the simulation results indicate that it has a better extraction effect, stronger anti-noise ability, faster processing speed, more flexible use, as well as can detect the edge direction information. In future work, the method proposed in this paper goes for further optimization both in terms of complementing the image detail information and further noise reduction. The excellent performance in fingerprint information extraction can apply this technology to similar medical or security applications. The extraction of information from color images can be extended to livestock quality control and other fields.

## Data Availability

The datasets used and/or analyzed during the current study are available. The data set in this article is from the Pascal Voc image database. http://host.robots.ox.ac.uk/pascal/VOC/voc2012/index.html.
